# Utilization of epinephrine-soaked gauzes to address bleeding from osteotomy sites in non-tourniquet total knee arthroplasty: a retrospective cohort study

**DOI:** 10.1186/s12891-020-03595-6

**Published:** 2020-08-25

**Authors:** Hongzhi Liu, Zhaohui Liu, Qidong Zhang, Wanshou Guo

**Affiliations:** 1 Beijing University of Chinese Medicine, 11 N 3rd Ring Rd E, Chaoyang District, Beijing, China; 2 Department of Orthopaedic Surgery, China-Japan Friendship Hospital, No. 2, Yinghuadong Road, Chaoyang District, Beijing, 100029 China; 3 Beijing Key Lab Immune-Mediated Inflammatory Diseases, China-Japan Friendship Hospital, No. 2, Yinghuadong Road, Chaoyang District, Beijing, 100029 China

**Keywords:** Epinephrine, Tourniquet, Total knee arthroplasty, Hemostatic effects, Hemodynamic

## Abstract

**Background:**

Reducing tourniquet inflation time is important because of the complications of tourniquet extensively used for the control of hemorrhage in total knee arthroplasty (TKA). Bleeding management is critical to acquire a relative bloodless arthrotomy interface for maximize cement fixation in non-tourniquet TKA. The purpose of this study was to investigate hemostatic and hemodynamic effects of epinephrine-soaked gauzes in cemented TKAs.

**Methods:**

A retrospective cohort study of 101 patients in two groups was performed. The first group (*n* = 51) underwent unilateral TKA with our procedures of epinephrine use, the second group (*n* = 50) had the same protocol with tourniquet and no epinephrine utilization. Surgical field visualization was assessed by grading scale for difficulty of intraoperative visualization due to blood and number of surgical field clearances. Perioperative blood loss was recorded. Hemodynamic parameters were observed in the epinephrine group.

**Results:**

There was statistically significant difference (*p* < 0.01) on surgeon-rated difficulty in visualization in the epinephrine group between before and after use of epinephrine, and no statistically significant difference (*p* = 0.96) between two groups before cementing. No statistically significant result on numbers of surgical field clearances between two groups (*p* = 0.25) was found. Epinephrine group showed significant difference in hidden blood loss compared with no epinephrine group (576.6 ± 229.3 vs 693.2 ± 302.9, respectively, *p* = 0.04). The hemodynamic effects of epinephrine may be under control.

**Conclusion:**

The procedure of epinephrine soaked gauzes, as a prudent adjunct, may be effective to reduce blood loss and obtain bloodless bone sections in non-tourniquet TKAs, regardless of hemodynamics.

## Background

Epinephrine is often used in local infiltration analgesia to reduce systemic absorption of the local anesthetic and prolong the analgesic effect in total knee arthroplasty (TKA) [1–3], and is also used to reduce perioperative blood loss [4, 5]. Epinephrine as a procoagulant has been demonstrated key activities associated with increased fibrinogen release, activation of fibrinogen receptors, and effects on other coagulation factors whose activities are mediated via beta-adrenergic receptor. Further research has showed that platelet count increases arise from splenic autotransfusion following low-dose epinephrine infusion via alpha-adrenergic receptors [6–8]. Consequently, the whole blood clotting time significantly decreases. Administration of epinephrine is controversial in joint replacement because of a composite of major cardiopulmonary complications including ventricular tachycardia, broken-heart syndrome, pulmonary edema, and intraventricular bleeding [9–11]. However, there has been little discussion about its hemostatic effect as a vasoconstrictor, and hemodynamic effect from administration of epinephrine in TKA procedure.

A pneumatic tourniquet is commonly used in TKA to improve visualization of the operative field, decrease intraoperative blood loss, and enhance the quality of cementation [12, 13]. Even in some tourniquet-free procedures, a tourniquet is also inflated during component cementation because some participating surgeons feel a tourniquet was necessary to minimize blood at the bone-cement interface and maximize fixation. However, acute hypotension and the ischemia-reperfusion (I-R) injury would occur when blood perfusion was re-established and release of metabolites from the ischemic limb and reduced cardiac preload after deflation of the tourniquet. And it would have a high risk of cardiac and cerebral micro emboli, a high risk of deep venous thrombosis, and an increased incidence of arterial thrombosis especially for the elderly undertaking TKA who have multiple comorbidities [14].

Therefore, for a tourniquet-free TKA protocol, our attempt was made to establish a set of intra-surgical procedures that can provide a satisfying visualization and a relatively bloodless bone interface for cementation with the topical hemostatic agent of epinephrine solution. Furthermore, epinephrine-related side effects associated with adverse cardiopulmonary complications may be fatal in rare cases and cautious hemodynamic monitoring is needed. The purpose of this study was to determine the hemostatic effect of epinephrine-soaked gauzes by comparing surgical field visualization between patients receiving epinephrine and those undergoing tourniquet. We hypothesized that epinephrine-soaked gauzes would be sufficient to reduce blood loss and obtain bloodless bone sections in patients undergoing non-tourniquet cemented TKA.

## Methods

### Patient selection

**Fig. 1:**
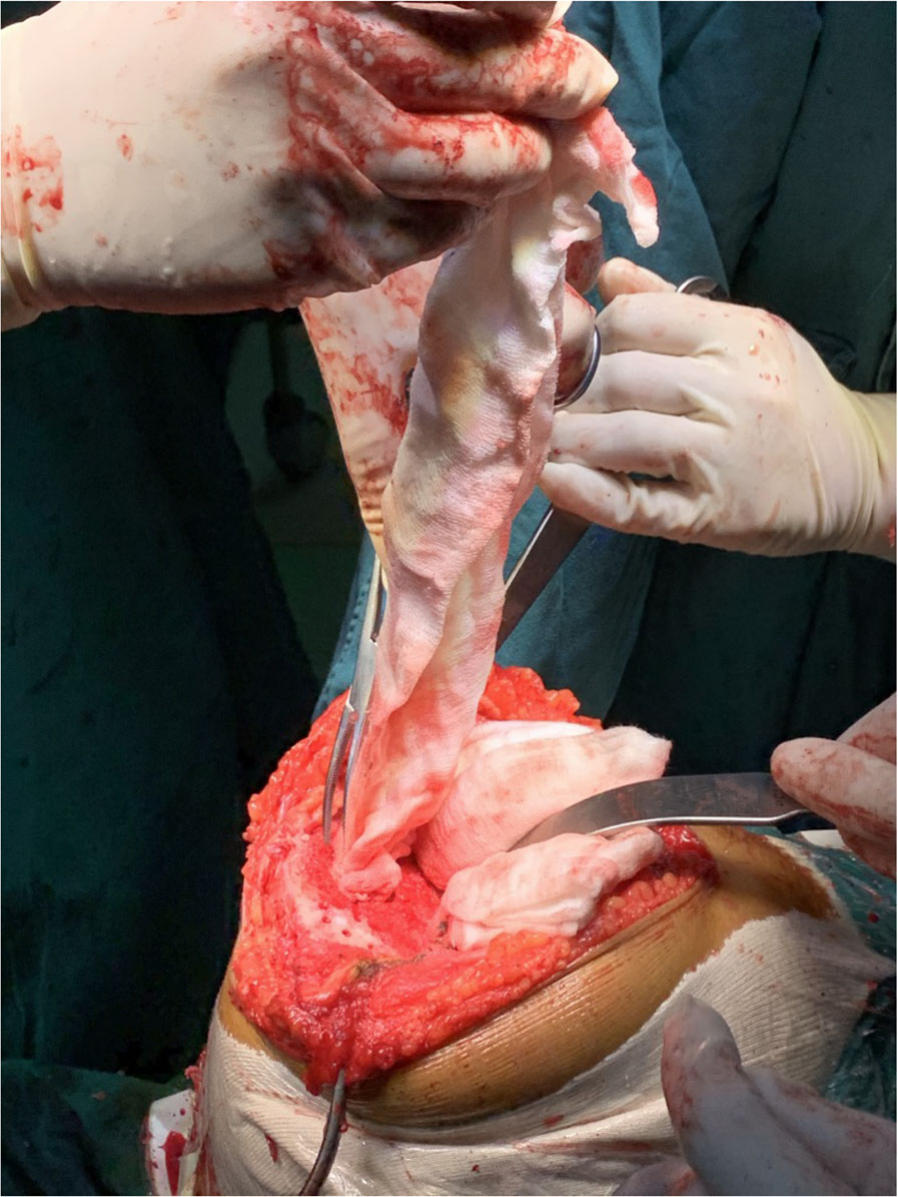
Epinephrine-soaked gauzes are covered and pressurized on osteotomy sites of femur and tibia to address bleeding

This study protocol has been approved by the IRB of the authors’ affiliated institutions. This is a retrospective cohort study of 143 patients who underwent a primary unilateral cemented TKA from January 1, 2018 to December 31, 2019 in our hospital. From the final analysis patients with prior surgery involving the femur or tibia, prior lower extremity fracture, coagulopathy and uncontrolled hypertension, history of myocardial infarction or stroke, and bilateral total knee arthroplasty were excluded. In the final analysis, a total of 101 patients fulfilled the study inclusion criteria and were available. The patients were analyzed in 2 groups. In the first group, one tourniquet was not used but prepared for bleeding out of control and gauzes soaked with 1-mg epinephrine mixed with 125-mL normal saline solution (dilution 1:125,000) were utilized to cover and pressurize for hemostasis on the osteotomized surface of the distal femoral and proximal tibial bones from the finish of bone resections to the start of cementing (Fig. 1). In the second group, one pneumatic tourniquet placed high on the thigh was inflated to 250 mmHg before the skin incision and deflated after the cement was completely polymerized. All other aspects of perioperative care were constant throughout the groups.

### Perioperative management

All the operations were performed with a fixed plant posterior-stabilized total knee prosthesis in a medial parapatellar approach. A general anesthetic with an adductor canal block was used for all the patients [15]. And appropriate perioperative parenteral antibiotics were administered for infection prophylaxis. High-pressure pulsatile lavage was used to clean the bone surfaces and soft tissues. Before wound closure, all knees received an intraarticular injection of a cocktail containing ropivacaine, morphine, and ketorolac to enhance postoperative analgesia without drain insertion. Elastic compressive dressing was also used. Each patient received the same perioperative regimen: tranexamic acid (TXA), pain control and rehabilitation. All patients received 1-g intravenous tranexamic acid 30 min before skin incision, and a solution of 1-g tranexamic acid in 50-mL normal saline solution was injected into knee articular cavity after closure of the capsule [16]. Multimodal postoperative pain management and accelerated physical therapy were performed as previously described. Low molecular weight heparin (enoxaparin, 3000 IU) was administered subcutaneously 12 h after the operation and was continued for 2 weeks for thrombosis prophylaxis. After recovery from anesthesia, quadriceps femoris muscle isometric contraction was immediately started, and rehabilitation began on the first postoperative day, including muscle power training.

### Outcome measurement

The primary outcomes of the study were surgical field visualization including surgeon-rated difficulty in visualization and number of surgical field clearances [17, 18]. Surgeon-rated difficulty in visualization prior to using epinephrine in Epinephrine group and before cementing in two groups, was graded (Grading scale for difficulty of intraoperative visualization due to blood: 0 No Difficulty; 1 Some difficulty, but did not affect the case; 2 Moderate difficulty; 3 Severe difficulty), and independently assessed by two senior authors (Z. L. and W. G.) at the end of the surgery; Number of surgical field clearances (excluding action of using epinephrine-soaked gauzes) was counted by another author (H. L.) in two groups over the period from the finish of bone cutting through the cement hardening. Any disagreement was resolved by a consensus among all authors.

Perioperative blood loss was the secondary outcome. Volumes of intraoperative blood loss, postoperative blood loss, blood transfusion, and hidden blood loss were recorded. The blood volume of each patient was calculated by a formula that consists of patient weight, height and sex [19]. The blood loss was calculated on the basis of the validated Gross formula [20]. Hematocrit (Hct) and hemoglobin (Hb) levels were determined preoperatively and on postoperative day 2. The intraoperative blood loss represented by the increased weight of the gauzes plus the volume in the aspirator bottle excluding rinse. The amounts of postoperative visible blood loss were calculated by weighing the dressings removed after the surgery. Intraoperative characteristics included the surgery time duration (beginning at incision until emergence from anesthesia), tourniquet time were noted. Meanwhile, in the epinephrine group, incidences of hypertension, hypotension and tachycardia were evaluated. Hemodynamic parameters at 5-min intervals for 15 min since timing of application of the epinephrine were recorded. At 4 weeks after surgery (the first postoperative follow-up visit), the incidence of symptomatic deep vein thrombosis (DVT), symptomatic pulmonary embolism (PE) and other complications, such as subcutaneous hematoma, skin complications (blisters, bruises, superficial and deep wound infection), myocardial infarction, and cerebrovascular accident, were assessed.

### Statistical analyses

Data were entered into an Access Case Report Form database and further analyzed by R software (version 3.6.3, The R Foundation, Vienna, Austria). Quantitative data are presented as the mean and standard deviation (SD); categorical variables are used as proportions. Differences in continuous variables between groups were evaluated with Independent-Sample T test or Mann-Whitney U test, depending on the distribution characteristics of the data. A chi-square test or Fisher exact test for difference in proportions was used to estimate the differences between groups in categorical variables. Values of *p* < 0.05 were considered to be statistically significant.

## Results

There were no significant differences (*p* > 0.05) found at baseline with regard to age, body mass index (BMI), sex, American Society of Anesthesiologists (ASA) class, mean starting hemoglobin, or preoperative diagnosis between the groups. In this study, 101 patients were available, as shown in Table 1: 51 in the epinephrine group, and 50 in the no epinephrine group. The tourniquet time for the tourniquet group was 61.1 min. The mean operating time for the epinephrine group (81.6 min) and the no epinephrine group (80.9 min) was not significantly different (*p* = 0.76). There was no significant between-group difference in follow-up duration (*p* = 0.41).

**Table 1:** Patient demographic characteristics

	Epinephrine group (*N* = 51)	No epinephrine group (*N* = 50)	*P* value
Age^a^ (yr)	67.1 (8.6)	66.8 (8.9)	0.88†
Sex^b^			0.34#
Male	4 (7.8%)	8 (16.0%)	
Female	47 (92.2%)	42 (84.0%)	
BMI^a^ (kg/m^2)	25.8 (3.2)	26.2 (3.4)	0.55†
Starting hemoglobin^a^ (g/dL)	131.8 (12.3)	132.4 (14.3)	0.22†
ASA classification^b^			0.32§
1 (normal, healthy)	20 (39.2%)	19 (38.0%)	
2 (mild, systemic disease)	30 (58.8%)	29 (58.0%)	
3 (severe systemic disease)	1 (2.0%)	3 (6.0%)	
Preoperative diagnosis^b^			0.99**
Osteoarthritis	50 (98.0%)	48 (96.0%)	
Rheumatoid arthritis	1 (2.0%)	2 (4.0%)	
Tourniquet time^a^ (min)	NA	61.1 (7.8)	
Operating time^a^ (min)	81.6 (12.1)	80.9 (9.3)	0.76†
Follow-up duration^a^ (day)	28.5 (2.5)	28.2 (2.6)	0.41†

^a^The values are given as the mean and the standard deviation

^b^The values are given as the number of patients, with the percentage in parentheses

†Significance was determined with use of Independent-Sample T test

§ Significance was determined with use of the Mann-Whitney U test

# Significance was determined with use of the chi-square test

**Significance was determined with use of the Fisher exact test

In terms of surgical field visualization, there was statistically significant difference (*p* < 0.01) on surgeon-rated difficulty in visualization in the epinephrine group between before and after use of epinephrine, and no statistically significant differences (*p* = 0.96) between two groups before cementing (Table 2). We also found no statistically significant result on numbers of surgical field clearances between two groups (*p* = 0.25) (Table 3).

**Table 2:** Surgeon-rated difficulty in visualization

	Before Epinephrine^a^		Before Cementing^a^		*P* value
	Epinephrine group	No epinephrine group	Epinephrine group	No epinephrine group	
	(*N* = 51)	(*N* = 50)	(*N* = 51)	(*N* = 50)	
0 No Difficulty	0 (0.0%)	NA	42 (82.3%)	41 (82.0%)	< 0.01§# 0.96§##
1 Some difficulty, but did not affect the case	6 (11.8%)	NA	8 (15.7%)	8 (16.0%)	
2 Moderate difficulty	12 (23.5%)	NA	1 (2.0%)	1 (2.0%)	
3 Severe difficulty	33 (64.7%)	NA	0 (0.0%)	0 (0.0%)	

^a^The values are given as the number of patients, with the percentage in parentheses

§Significance was determined with use of the Mann-Whitney U test

# P value was calculated for difference in Epinephrine group between before and after use of epinephrine

## *P* value was calculated for difference between Epinephrine and No Epinephrine groups before cementing

**Table 3:** No of surgical field clearances

	Epinephrine group^a^ (*N* = 51)	No epinephrine group^a^ (*N* = 50)	*P* value
None	17 (33.3%)	21 (55.3%)	0.25§
1–3 cycles	29 (56.9%)	27 (48.2%)	
> 3 cycles	5 (9.8%)	2 (28.6%)	

^a^The values are given as the number of patients, with the percentage in parentheses

§Significance was determined with use of the Mann-Whitney U test

Table 4 shows that the epinephrine group had significantly less hidden blood loss (*p* = 0.04) than the no epinephrine group. There was no between-group difference in the calculated blood loss (*p* = 0.26), intraoperative blood loss (*p* = 0.87). Two patients in the no epinephrine group, and 2 patients in the epinephrine group received a blood transfusion.

**Table 4:** Comparison of differences in blood loss

	Epinephrine group (*N* = 51)	No epinephrine group (*N* = 50)	*P* value
Calculated blood loss^a^# (mL)	735.9 (293.7)	847.7 (333.8)	0.26†
Intraoperative blood loss^a^ (ml)	175.3 (83.7)	170.5 (80.9)	0.87†
Hidden blood loss^a^ ## (ml)	576.6 (229.3)	693.2 (302.9)	0.04†
Transfusion^b^			0.99§
None	49 (96.1%)	48 (96%)	
2 unit	0 (0.0%)	0 (0.0%)	
4 units	2 (3.9%)	2 (4.0%)	

^a^The values are given as the mean and the standard deviation

^b^The values are given as the number of patients, with the percentage in parentheses

†Significance was determined with use of Independent-Sample T test

§significance was determined with use of the Fisher exact test

# PBV (Patient’s blood volume) = k1 x height^3 (m) + k2 x weight (kg) + k3, where k1 = 0.3669, k2 = 0.03219, and k3 = 0.6041 for men; and k1 = 0.3561, k2 = 0.03308, and k3 = 0.1833 for women; Total blood loss = PBV x (preoperative Hct – postoperative Hct)

## Hidden blood loss = Total blood loss – Intraoperative blood loss + Allogeneic blood transfusion

**Fig. 2:**
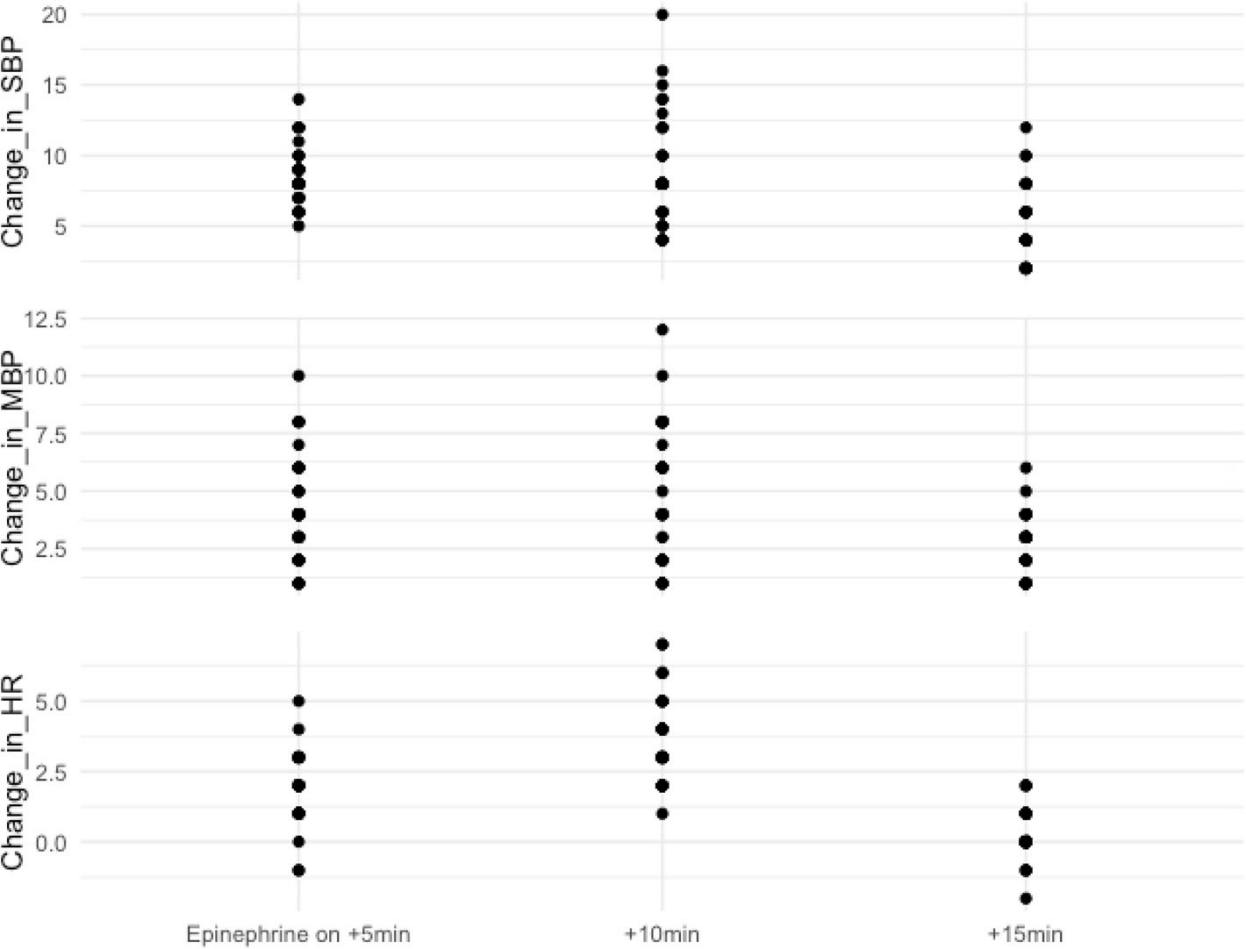
Hemodynamic parameters at 5-min intervals for 15 min since timing of application of the epinephrine. HR, heart rate; MBP, mean blood pressure; SBP, systolic blood pressure

The incidence of hemodynamic instability after application of epinephrine, including hypertension, hypotension, and tachycardia, is shown in Table 5. Figure 2 presents the changes in hemodynamic parameters, including systolic blood pressure (SBP), mean blood pressure (MBP), and heart rate (HR), at 5-min intervals from timing of use of the epinephrine to 15 min following that.

**Table 5:** Incidences of hemodynamic instabilities after application of the epinephrine

	Epinephrine group^a^ (*N* = 51)
Systolic BP > 190 mmHg or mean BP > 140 mmHg	0 (0.0%)
Systolic BP > 140 mmHg or mean BP > 110 mmHg	6 (11.8%)
Systolic BP increase of 20% from baseline	4 (7.8%)
Systolic BP < 90 mmHg or mean BP < 60 mmHg	7 (13.7%)
Tachycardia (heart rate > 100 beats per minute)	1 (2.0%)

^a^The values are given as the number of patients, with the percentage in parentheses. *BP* Blood pressure

Postoperative deep venous thrombosis and other complication percentages in each group are presented in Table 6. Two patients in in the no epinephrine group developed skin blisters and 2 other patients did bruises. No patients had superficial or deep wound infection, myocardial infarction, or cerebrovascular accident postoperatively.

**Table 6:** Complications

	Epinephrine group^a^ (*N* = 51)	No epinephrine group^a^ (*N* = 50)	*P* value
**Deep venous thrombosis or pulmonary embolism**			**0.24§**
None	51 (100%)	48 (96%)	
Deep venous thrombosis	0 (0%)	2 (4%)	
Pulmonary embolism	0 (0%)	0 (0%)	
**Other complications**^b^			**0.52§**
None	49 (96.1%)	45 (90.0%)	
Subcutaneous hematoma	1 (1.9%)	1 (2.0%)	
Skin complications			
Blisters	0 (0.0%)	2 (4.0%)	
Bruises	1 (1.9%)	2 (4.0%)	

^a^The values are given as the number of patients, with the percentage in parentheses

^b^No patients in the data set were recorded as having superficial infection, deep infection aseptic loosening, myocardial infarction, or cerebrovascular accident as other complications

§Significance was determined with use of the Fisher exact test

## Discussion

We found that ideal bloodless visualization of the operative field was obtained in the epinephrine group, comparing that in epinephrine group between before and after use of epinephrine and in two groups prior to cementing. We also found that the use of epinephrine resulted in reducing postoperative hidden blood loss significantly and hemodynamic parameters fluctuated under control in patients receiving epinephrine.

In terms of topical control of blood loss in TKA, epinephrine solution, TXA, and many hemostatic gels and powders have been advocated to address bleeding recently [21]. The combined administration of low-dose epinephrine and TXA also demonstrated an increased effect in reducing perioperative blood loss and the inflammatory response [22]. All of these methods have been used to reduce the need for allogenic transfusion. However, none of these methods was used to address bleeding from osteotomy sites for visualization and cementing.

Vasoconstriction effect of epinephrine is complex, and vasoconstriction intensity differs depending on vessel type: arteries, arterioles, precapillary sphincters, capillaries, venules, and veins [23]. It is conceivable that the use of vasoconstrictor epinephrine might predispose to delayed intraoperative bleeding, by temporarily blocking vessels that later start bleeding when the initial vasoconstrictor effect has passed. Though epinephrine’s maximal effect on arterial vasoconstriction may work at 7 to 10 min, it takes considerably longer for a new local equilibrium to be obtained with regard to hemoglobin quantity. If optimal visualization and fixation are desired, the ideal time for cement hardening should be the time when local hemoglobin concentration is lowest [24, 25]. Therefore, it is sufficient for fully cementing all areas of contact of the tibia and femur by preparing cement, applying it to both the bone and the component surfaces, then holding components carefully in place until the cement has completely polymerized. On top of that, there was no evidence of any rebound bleeding in the postoperative period in any of our patients, as an indication in the nearly equal volume of intraoperative blood loss that occurred. This finding also suggests that the use of epinephrine does not increase the risk of intraoperative rebound bleeding with good hemostasis for the occurrence of the roughly same volume of intraoperative blood loss.

The present study showed topical use of epinephrine-soaked gauzes induced a significant reduction of perioperative hidden blood loss compared with utilization of tourniquet. Epinephrine as a platelet-stimulating agent can cause aggregation of human platelets through alpha-adrenergic mechanisms [8]. It can explain the effectiveness of this procoagulant in decreasing postoperative blood loss because of its hemostatic effect [26]. Moreover, insufficient intra-operative hemostasis under tourniquet and tourniquet release further promote local fibrinolysis, which may result in further blood loss [27].

This method was characterized by the absence of complications and adverse reactions associated with epinephrine. Hemodynamic parameters in our study fluctuated under control in patients receiving epinephrine. Peak changes in HR, MBP, SBP were observed to reached 10 min following the beginning of the epinephrine use, and the original values were decreased to at approximately 15 min from start. Tissue ischemia, infection and skin necrosis were also not detected in the epinephrine group.

This study had several limitations. First, regarding the concentration and technique of epinephrine solution, there is no consensus. To our knowledge, there is not a single study with outcome measurements that epinephrine is used for hemostasis in the osteotomized sites. A body of previous studies have reported subcutaneous injection of epinephrine solutions in concentrations up to 1:50,000 with good effect in burn and hand surgery [28–31], and even higher concentration has been safely used in other forms of surgery [32, 33]. We empirically prepared an epinephrine solution at a concentration of 1:125,000 which was higher than that in some orthopaedic surgery, on the basis of consideration that effective drug concentration was longer for cement hardening before it wore off. Meanwhile, the tumescent technique as it applies to suction lipectomy of plastic surgery has been more extensively studied, but these data are not directly applicable to orthopaedic cases as much of the epinephrine is used in bone cut [34, 35]. Hence, we attempted to utilize the technique of epinephrine, soaking not infiltrating, on the osteotomized surfaces, because tumescent or infiltrating technique was abandoned for such adverse effects of epinephrine as delaying wound healing, increasing the risk of infection, and compromising flap survival. Then, the incidence of hemodynamic instability may have been obscured because we merely investigated hemodynamic parameters at 5-min intervals only for 20 min since application of the epinephrine and bone cement may adversely affect hemodynamics. Furthermore, short-, medium-, and long-term outcomes outcome following TKA is needed to evaluate whether the epinephrine procedure will compromise prothesis survival or not. Additionally, since we did not have a control group without a tourniquet and epinephrine both together, hence we may not assess the net effect of epinephrine regardless of physiological hemostasis, if any. Finally, we failed to find any significant differences in the incidence of postoperative complications, this might be due to a relatively small sample size or lack of clinical implication. Further large-scale studies on high-risk patients are needed to assess the association between intra- and postoperative cardiovascular complications and the use of epinephrine.

## Conclusion

The procedure of epinephrine-soaked gauzes can result in an effective form of addressing blood oozing from cut bone planes, while it does not lead to apparent hemodynamic oscillations and vigilant hemodynamic monitoring is a necessity. The utilization of epinephrine-soaked gauzes seems to have an advantage in terms of great potential for a complete tourniquet-free procedure, regardless of hemodynamics.

## Data Availability

The datasets used and/or analyzed during the current study are available from the corresponding author on reasonable request.

## Abbreviations

ASA: American Society of Anesthesiologists
BMI: Body mass index
BP: Blood pressure
CTPA: Computed tomography pulmonary angiogram
DVT: Deep vein thrombosis
Hb: Hemoglobin
Hct: Hematocrit
HR: Heart rate
MBP: Mean blood pressure
PE: Pulmonary embolism
SBP: Systolic blood pressure
TKA: Total knee arthroplasty
TXA: Tranexamic acid
